# LIVING IN A DOLL HOUSE: A CASE REPORT AND LITERATURE REVIEW OF REDUPLICATIVE PARAMNESIA

**DOI:** 10.1192/j.eurpsy.2023.1976

**Published:** 2023-07-19

**Authors:** J. M. Pinto, I. Faria, C. P. Gouveia, J. Andrade

**Affiliations:** Psychiatry, Coimbra Hospital and University Center, Coimbra, Portugal

## Abstract

**Introduction:**

Reduplicative paramnesia (RP) is a very rare content-specific delusional misidentification syndrome (DMS). RP entails the delusion that a place, an object, or an event has been duplicated or exists in two different places at the same time. RP is thought to result from an organic rather than psychiatric cause distinguishing it from other DMS. It has been suggested that damage to the right frontal and temporal lobe plays a crucial role, although other areas involved in visuospatial processing have also been reported.

**Objectives:**

The aim of this study is to review the literature and report a clinical case of RP.

**Methods:**

We describe a case of an 81 year old woman admitted in a Neurology ward, with a 2 week clinical presentation of temporo-spatial disorientation, behavioural changes, persecutory delusions and reduplicative paramnesia phenomena concerning her house. She had previous history of a stroke 3 years prior to admission and, about one year before, the patient also started to present cognitive decline in the context of Parkinson’s dementia. One month before admission, treatment with Rotigotine was started and later suspended when the aforementioned clinical manifestations started. Upon admission it was diagnosed an urinary tract infection and treatment with antibiotics was started. Two days afterwards, the patient recovered orientation and her usual behaviour, but persecutory delusions and RP persisted. She then started treatment with low dose Olanzapine. Following 2 weeks of treatment the psychotic symptoms fully remitted, including RP.

**Results:**

We underline CT-scan and EEG relevant findings upon admission. In the CT-scan sequelar lesions in left frontoparietal junction, right posterior frontal cortex, left inferior occipital cortex, bilateral cerebellar hemispheres, left caudate nucleae and thalamus were identified. The EEG showed a preserved posterior alpha rhythm associated with slow discontinuous right temporal and mainly left parieto-temporo-occipital activity, indicating dysfunction in these locations.

**Conclusions:**

In line with literature our patient had lesions in the right frontal and temporal lobe. She also presented lesions in other areas involved with visuospatial processing. Particularly the involvement of the left hemisphere reported in our case seems to be an exception. Other factors potentially played a role triggering this episode, namely the cognitive compromise due to dementia interposed with infectious disease, and the rotigotine treatment as well. Another aspect worth mentioning in our case was the remission of symptoms with the use of Olanzapine, even though only a few cases in literature have fully remitted with treatment with antipsychotics.

**Disclosure of Interest:**

None Declared

